# The Influence of Short-Video Usage on Prospective Memory Under Different Cue Type Conditions

**DOI:** 10.3390/bs16060904

**Published:** 2026-06-02

**Authors:** Kaiyue Zhai, Yunfei Guo, Li Wang

**Affiliations:** 1 Eurasian International College of Henan University, Henan University, Kaifeng 453007, China; 2 Henan Key Laboratory of Psychology and Behavior, Henan University, Kaifeng 453007, China

**Keywords:** short-form videos, focality, prospective memory, attention, cues

## Abstract

Short-form videos are online content designed for fragmented mobile viewing, with typical durations of 3 s to 5 min. This consumption pattern disrupts attention—a critical resource for prospective memory (PM)—the ability to remember intended future actions. Therefore, short-form video use may negatively affect PM performance. This study examined this effect and its underlying cognitive mechanisms through two experiments. Study 1 adopted a 2 (group: experimental group, control group) × 2 (cue type: focal, non-focal) between-subjects design, recruiting 106 college students to explore the impact of short-form video use on PM with different cue types. The experimental group watched short videos before the experiment, while the control group viewed long videos. Study 2 further reduced the number of cues for the non-focal PM task, recruiting 64 college students to examine the effect of short-form videos on non-focal PM under a single-cue condition. The results showed that prolonged viewing of short-form videos significantly reduced the accuracy of both focal and non-focal PM, but had no significant impact on the ongoing task. When the number of PM cues was reduced to one, the detrimental effect of short-form videos on non-focal PM weakened. These findings indicate that the impairment of PM by short-form videos depends on the attentional demands of the task, and a single cue can resist such impairment by reducing attentional consumption.

## 1. Introduction

Driven by the dual forces of the mobile Internet and intelligent terminals, the short-form video industry has experienced explosive growth worldwide. Such content is mainly characterized by short duration, tight rhythm, and fragmentation (Alruwaili, 2025; Nguyen et al., 2025). With the accelerating pace of modern social life, people have accumulated a large amount of fragmented time in scenarios such as commuting and queuing. Short-form videos typically range from 3 s to 5 min, which perfectly meets the demand for acquiring information or entertainment using fragmented time. Users do not need to invest continuous blocks of time and can quickly browse content through simple sliding operations. It is this high matching between technical characteristics and user needs that has made short-form video browsing evolve into a mainstream leisure and entertainment method. However, prolonged viewing of short-form videos can interfere with abilities such as attention and memory (Alruwaili, 2025), and prospective memory (PM). The frequent switching and fragmented content presentation of short videos can alter our attention patterns, leading to an inability to sustain focused attention on specific stimuli (Chiossi et al., 2023). However, PM refers to the memory of remembering to perform planned actions in specific situations (Einstein & McDaniel, 1990). Monitoring for PM cues typically requires sustained attention, and attentional disruptions can lead to more PM omission errors (Guo et al., 2025). Therefore, short-video use can interfere with PM, and such interference can seriously impact our quality of life when it leads to everyday failures like forgetting to turn off a gas stove or to take medication, which pose safety and health risks. This makes it highly significant, practically, to examine the relationship between short-form video use and PM.

### 1.1. Attention Residue Theory and Short Video Usage

The attention residue theory points out that when an individual switches from one task to another, the cognitive processing of the previous task does not stop immediately but continues to exist in the form of attention residue, thereby occupying limited cognitive resources and making it difficult for subsequent tasks to obtain sufficient attentional support (Leroy, 2009; Leroy & Glomb, 2018). Short-video use affects multiple cognitive functions. For instance, it impairs sustained attention (Lin et al., 2024), and long-term exposure to short videos also damages executive function (Xiao et al., 2026). The successful implementation of PM heavily relies on cognitive abilities such as attention and executive function (Ball et al., 2022; Mahy & Moses, 2011). Accordingly, short-video use tends to interfere with PM. On this basis, Chiossi et al. (2023) further proposed that after watching short-form videos, individuals’ attention may still linger on the video content they just watched, and this continuous cognitive activation will weaken their ability to process information related to PM tasks. At the same time, a growing body of research evidence shows that the frequent switching and high-stimulation characteristics of short-form videos are likely to cause repeated interruptions of sustained attention (Yan et al., 2024) and reduce individuals’ attention stability and concentration in subsequent tasks (Lin et al., 2024). These factors collectively further aggravate the insufficient attentional processing of PM tasks, which may have a negative impact on PM task performance.

### 1.2. Mechanisms of Prospective Memory Impairment in Short Video Users

Chiossi et al. (2023) randomly assigned participants to four groups: Rest, Twitter, YouTube, and TikTok. The ongoing task employed a lexical decision task. The results showed that only the TikTok group exhibited a significant decline in PM accuracy, while the other three groups showed no significant changes in PM performance. They argued that the frequent context switching in short-form videos impairs intention maintenance in the PM task, which in turn leads to the detrimental effect of short-form video use on PM. Barton and Smyth (2025) further manipulated the frequency of video content switching, dividing participants into a control group, a limited switching group, and an unlimited switching group, involving the platforms TikTok, YouTube Shorts, and Instagram Reels. The results showed that only the unlimited switching group experienced a significant decline in PM accuracy, directly confirming that frequent short-form video content switching is the core mechanism underlying PM decline. These findings highlight the critical role of attentional resources in short-form video-related PM impairment, which makes it necessary to distinguish between different PM subtypes based on their attentional demands. Both studies attribute PM impairment to attentional resource depletion caused by content switching, but they used different types of PM tasks that vary in their attentional demands. According to cue types, PM can be divided into focal PM and non-focal PM. The identification of focal cues highly overlaps with the processing of the ongoing task, so it has a low dependence on attention. However, the identification of non-focal cues by individuals has a low degree of overlap with the ongoing task and is highly dependent on attention (Altgassen et al., 2009; Scullin et al., 2010). Non-focal PM requires self-initiated attentional resources for cue monitoring. Like time-based PM, which refers to the memory of completing planned tasks at appropriate future time points or within specific time periods, it is associated with relatively high task difficulty (Guo et al., 2025). Specifically, Chiossi et al. (2023) used a non-focal PM task, while Barton and Smyth (2025) adopted a focal PM task. According to cue types, PM can be divided into these two main categories. Focal PM relies primarily on automatic retrieval processes and requires minimal attentional resources, while non-focal PM requires sustained attentional monitoring and is thus more vulnerable to resource depletion. This methodological difference may account for some of the variation in effect sizes observed across studies. Therefore, it is essential to explore whether the impairing effect of short-form video use on PM is affected by cue focality under identical experimental tasks. According to the attention residue theory, after excessive viewing of short-form videos, individuals’ attention will partially remain on the previously watched short-form video content, which will impair the performance of non-focal PM that is highly dependent on attention, but will not damage focal PM that has a low dependence on attention. The first purpose of this study is to verify the attention residue theory by focusing on whether the impairment of PM by short-form videos is affected by cue focality.

Although non-focal PM is highly dependent on attention, the number of cues may affect the degree of attention dependence of non-focal PM. Compared with multiple PM cues, a single PM cue reduces the attentional consumption used by individuals for PM cue confirmation during intention maintenance, and it is also easy to form an automatic connection between cues and behaviors (Einstein & McDaniel, 2010; Rothen & Meier, 2014). Therefore, with the reduction in the number of cues, the attentional demand of non-focal PM tasks may also decrease. Although the attention residue theory holds that prolonged viewing of short-form videos will cause part of the attention to remain on the short-form video content, leading to insufficient processing of subsequent tasks (Chiossi et al., 2023; Leroy, 2009), PM tasks with a single cue have reduced attentional demand, so the impairment of PM with a single cue by short-form videos will decrease. Therefore, the second purpose of this study is to focus on whether the impairment of PM by short-form videos will weaken under the condition of a single cue.

### 1.3. The Present Study

This study aimed to verify whether the attention residue theory could reasonably explain the impairment of PM by short-form videos by manipulating cue focality and the number of cues to change the attentional demand of PM tasks. Among them, Study 1 focused on the impact of short-form videos on PM under different cue focality conditions. Study 1 adopted a multi-cue PM task, which imposes high demands on attention. When the PM cues are reduced to a single one, the impairing effect of short-video use on PM may vanish. Accordingly, Study 2 further explored whether the adverse impact of short videos on single-cue PM would weaken. According to the attention residue theory, this study hypothesized that short-form videos only impaired non-focal PM performance, and under the single-cue condition, the impairment of PM by short-form videos would weaken. This study is the first to systematically verify the attention residue theory, which helps us deeply understand the processing mechanism of PM impairment by short-form videos.

## 2. Study 1

### 2.1. Participants

This study adopted the G*Power 3.1 to calculate the minimum sample size for a 2 (group) × 2 (cue type) analysis of variance. Based on the results of previous studies (Chiossi et al., 2023; Leroy, 2009), the effect size ranged from large to medium. This study set 1-β = 0.80 and α = 0.05. If it was a medium effect size (*f* = 0.25), the sample size would be equal to 128. If it was a large one (*f* = 0.40), the sample size would be equal to 52. Therefore, it is reasonable that the final number of recruited participants is between 52 and 128. Since this study focuses on the impact of short-form videos on PM, only participants who reported watching short-form videos for 0.5 to 3 h daily were eligible to take part in this study. This criterion is adopted to exclude those who basically do not watch short-form videos and those who may have short-form video addiction. We ultimately recruited 120 college students (46 males) aged between 19 and 25, who were equally divided into the focal control group, focal experimental group, non-focal control group, and non-focal experimental group. Participants with ongoing task accuracy lower than the random level and those who completely forgot the PM task after the experiment were excluded (14 participants), and finally 106 valid participants remained, including 26 in the focal control group (*M*_age_ = 21.08, *SD* = 1.79), 24 in the focal experimental group (*M*_age_ = 21.25, *SD* = 1.70), 27 in the non-focal experimental group (*M*_age_ = 21.59, *SD* = 1.65), and 29 in the non-focal control group (*M*_age_ = 20.79, *SD* = 1.84). All participants were required to sign an informed consent form before the experiment. This study was approved by the Ethics Committee of the Biomedical Research Ethics Committee of Henan University (HUSOM2025-779). This study was not pre-registered because, at the time of its design and data collection, pre-registration was not a standard practice in our research group for this type of experimental work. We acknowledge that this represents a limitation, as pre-registration would have strengthened the confirmatory nature of our analyses.

### 2.2. Experimental Procedure

Before the experiment, participants in the experimental group were required to open their commonly used short-form video APP (e.g., TikTok 34.0) and watch videos for 40 min, with the video content recommended by the APP based on the participants’ previous browsing history. The control group opened a video software APP they had used (e.g., Youku 11.1) and could watch 40 min of long-form videos according to their own preferences. Sustained engagement in an activity for more than 30 min is required to achieve a state of deep immersion (Chen et al., 2024), accordingly, the present study set the duration of short-video viewing to 40 min. To avoid the possibility that the control group frequently changing long-form videos might produce similar effects to short-form videos, we required the control group not to switch video content within every 10 min. After watching the videos, all participants were required to report their overall preference for the watched videos and their average daily video viewing time in daily life.

**Figure 1:**
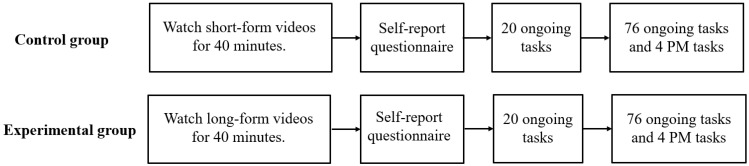
Experimental procedure flowchart.

**Figure 2:**
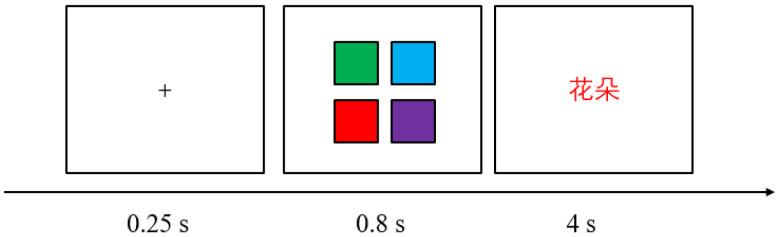
Experimental task flow chart. Among them, the focal PM task is to judge whether the color of the word is red, and the non-focal PM task is to judge whether the word is one of the two cue words.

After completing the self-report, the experiment entered the practice phase. At the beginning of the practice phase, instructions for the ongoing task were presented. The ongoing task was a color judgment task. Each ongoing task started with a plus sign fixation point (0.25 s), followed by four color blocks (0.8 s), and finally a word. Participants needed to judge whether the font color of the current word had appeared in the previous color blocks. If it had appeared, press the J key; otherwise, press the F key. If the participant did not respond at all, the word would disappear after 4 s. Participants were required to perform 20 ongoing tasks in the practice phase, with an accuracy exceeding 0.7 (see Figure 1 and Figure 2).

At the beginning of the formal experiment phase, instructions for the PM task were presented. The non-focal PM task was to directly press the spacebar when participants encountered either of the words “平均” (average) or “仪式” (ritual). In the focal PM task, participants were instructed to press the spacebar whenever they encountered words presented in red font. All words are high-frequency disyllabic words selected from the Modern Chinese Frequency Dictionary, with their frequencies ranging from 1000 to 5000. The formal procedure included a total of 76 ongoing tasks and four PM tasks. The focal PM cue appeared four times in total, while each non-focal PM cue appeared twice. The presentation order of the two non-focal PM cues was pseudorandom. Half of the participants received the sequence in ABAB order, and the other half received it in BABA order. There were at least 15 ongoing tasks between each two PM tasks.

### 2.3. Results of Study 1

SPSS 19.0 was used for 2 (group: experimental group, control group) × 2 (cue type: focal, non-focal) analysis of variance in Study 1.

Average daily short-form video viewing time: The results of the analysis of variance showed that the main effects of group and cue type, as well as the interaction between them, were not significant, *p* > 0.05 (see Table 1).

Preference for video content: The results of the analysis of variance showed that the main effects of group and cue type, as well as the interaction between them, were not significant, *p* > 0.05 (see Table 1).

PM accuracy: A 2 (group: experimental vs. control) × 2 (cue type: focal vs. non-focal) between-subjects ANOVA revealed a significant main effect of group, *F*(1, 102) = 6.38, *p* = 0.013, *η_p_*^2^ = 0.06, indicating a small-to-medium effect size. Participants in the experimental group (*M* = 0.47, *SD* = 0.31) exhibited significantly lower PM accuracy than those in the control group (*M* = 0.62, *SD* = 0.32).

**Figure 3:**
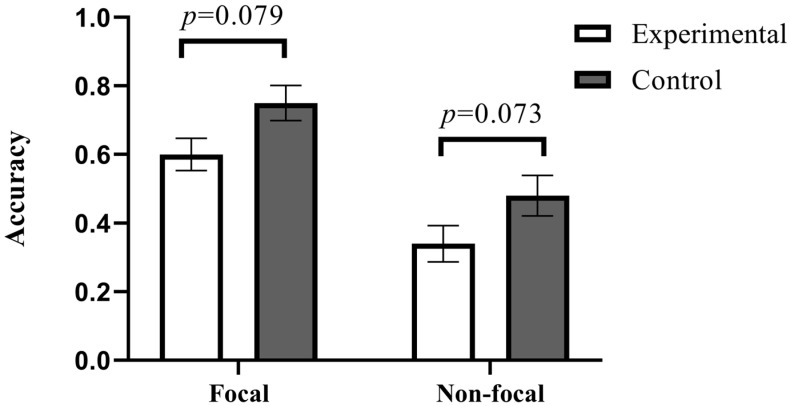
The accuracy of prospective memory in Study 1. Experimental = experimental group, Control = control group, an asterisk denotes significance at *p* < 0.05.

Consistent with theoretical expectations based on the multiprocess theory, there was a robust main effect of cue type, *F*(1, 102) = 21.81, *p* < 0.001, *η_p_*^2^ = 0.17, representing a medium-to-large effect size. As predicted, non-focal PM accuracy (*M* = 0.41, *SD* = 0.32) was significantly lower than focal PM accuracy (*M* = 0.67, *SD* = 0.28) across both experimental conditions. The interaction between group and cue type was not statistically significant, *F*(1, 102) = 0.12, *p* = 0.732, *η_p_*^2^ < 0.01, indicating that the detrimental effect of short-form video viewing was comparable for both focal and non-focal PM tasks (see Table 2 and Figure 3). To further confirm whether the experimental and control groups indeed differed under the focal and non-focal conditions, we conducted a simple-effect test. The results revealed that in the focal condition, the experimental group’s accuracy tended to be lower than that of the control group, *F*(1, 102) = 3.14, *p* = 0.079, *η_p_*^2^ = 0.03; in the non-focal condition, the experimental group’s accuracy also tended to be lower than that of the control group, *F*(1, 102) = 3.25, *p* = 0.073, *η_p_*^2^ = 0.03. This indicates that short-form video use tended to impair PM performance under both focal and non-focal conditions, and the effect size was small to medium.

PM reaction time: The ANOVA revealed a significant main effect of cue type, *F*(1, 102) = 6.38, *p* = 0.013, *η_p_*^2^ = 0.06 (small-to-medium effect). Participants responded faster to focal PM cues (*M* = 1028 ms, *SD* = 292 ms) than to non-focal PM cues (*M* = 1171 ms, *SD* = 413 ms), which is also consistent with the higher processing demands of non-focal tasks. No significant main effect of group or interaction effect was observed, *p*s > 0.05.

Ongoing task accuracy: A significant main effect of cue type was found, *F*(1, 102) = 6.77, *p* = 0.011, *η_p_*^2^ = 0.06 (small-to-medium effect). Ongoing task accuracy was higher in the non-focal PM condition (*M* = 0.81, *SD* = 0.08) than in the focal PM condition (*M* = 0.77, *SD* = 0.09). No significant main effect of group or interaction effect was detected, *p*s > 0.05.

Ongoing task reaction time: The main effect of group was marginally significant, *F*(1, 102) = 3.20, *p* = 0.077, *η_p_*^2^ = 0.03. The experimental group showed a tendency toward faster response speed than the control group, with the effect size ranging from small to moderate. The main effect of cue type and the interaction between the two variables were both non-significant, *p*s > 0.05 (see Table 2).

### 2.4. Discussion of Study 1

The results of Study 1 showed that there were no significant differences between the experimental group and the control group in terms of average daily short-form video viewing time and preference for video content, which excluded the interference of previous video-viewing experience and preference for video content. The PM accuracy of the experimental group showed a tendency to be lower than that of the control group, indicating that short-form video viewing does impair both types of PM performance. Under different cue-type conditions, the ongoing task reaction speed in the experimental group tended to be faster than that in the control group. This indicates that short video use reduces task processing time and attenuates attention consumption during the intention retention interval. In conclusion, Study 1 generally found that short-form videos impair PM performance, and the impairment is not affected by cue type.

However, a single PM cue is not only easy to identify but also easy for forming an automatic connection between cues and behaviors (Einstein & McDaniel, 2010; Rothen & Meier, 2014), which can reduce the attentional consumption of PM tasks. According to the attention residue theory, under the single-cue condition, the impairment of PM by short-form videos may weaken. Study 1 showed that short-video viewing impaired both focal and non-focal PM, hinting that bottom-up spontaneous retrieval may also be affected. To test this, Study 2 used a single non-focal cue to increase reliance on spontaneous retrieval and reduce monitoring demands. If the impairment weakened, short videos would mainly disrupt monitoring; if it persisted, spontaneous cue identification is also impaired. Study 2 can not only further test existing theories but also possesses practical significance. For instance, if single-cue PM tasks can mitigate the detrimental effect of short-video use on PM performance, then in daily life, multi-cue PM tasks can be decomposed into several single-cue PM tasks to alleviate such impairing effects.

## 3. Study 2

### 3.1. Participants

This study used the same sample size estimation method and recruitment criteria as Study 1, and ultimately recruited 70 college students (28 males) aged between 19 and 25, who were equally divided into the control group and the experimental group. Participants with ongoing task accuracy lower than the random level and those who completely forgot the PM task after the experiment were excluded; finally, 64 valid participants remained, including 32 in the control group (*M*_age_ = 21.00, *SD* = 2.01) and 32 in the experimental group (*M*_age_ = 21.22, *SD* = 2.39). All participants were required to sign an informed consent form before the study.

### 3.2. Experimental Procedure

The experimental procedure was basically the same as that of Study 1. The difference lies in that only one of the two PM cues from Study 1 was adopted in the experimental procedure. In each group, half of the participants were assigned “平均” (average) as the PM cue word, while the other half were assigned “仪式” (ritual) as the PM cue word. There were at least 15 ongoing tasks between any two consecutive PM cues. The formal procedure included a total of 76 ongoing tasks and 4 PM tasks.

### 3.3. Results of Study 2

SPSS 19.0 was used for one-way analysis of variance in Study 2.

Average daily short-form video viewing time: the results of the analysis of variance showed that there was no significant difference in the average daily short-form video viewing time between the experimental group and the control group, *p* > 0.05 (see Table 3).

Preference for video content: the results of the analysis of variance showed that there was no significant difference in the preference for video content between the experimental group and the control group, *p* > 0.05 (see Table 3).

**Figure 4:**
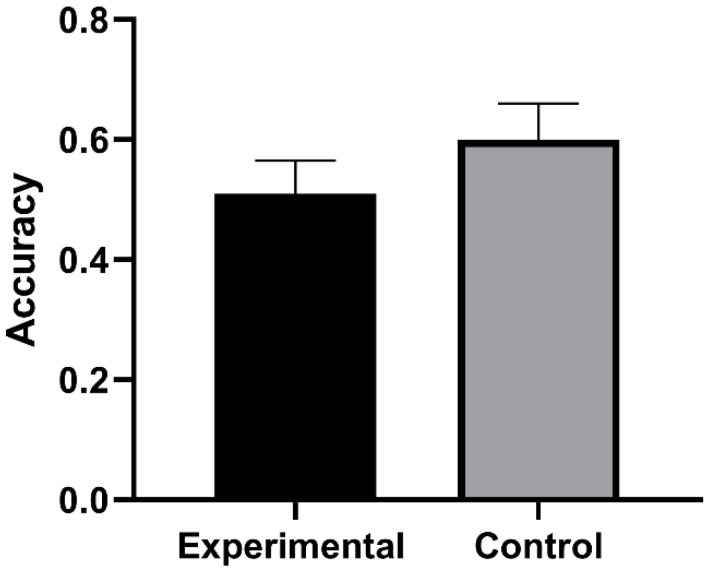
The accuracy of prospective memory in Study 2. Experimental = experimental group, Control = control group.

PM accuracy and reaction time: a one-way ANOVA revealed no significant difference in PM accuracy between the experimental group (*M* = 0.51, *SD* = 0.34) and the control group (*M* = 0.60, *SD* = 0.33), *F*(1, 62) = 1.12, *p* = 0.294, *η_p_*^2^ = 0.02. Similarly, no significant difference was found in PM reaction time between the two groups, *F*(1, 62) = 1.87, *p* = 0.176, *η_p_*^2^ = 0.03 (see Table 4 and Figure 4).

Ongoing task accuracy and reaction time: There were no significant differences in ongoing task accuracy (experimental: *M* = 0.79, *SD* = 0.08; control: *M* = 0.81, *SD* = 0.09), *F*(1, 62) = 0.89, *p* = 0.349, *η_p_*^2^ = 0.01, or reaction time (experimental: *M* = 1011 ms, *SD* = 254 ms; control: *M* = 1019 ms, *SD* = 215 ms), *F*(1, 62) = 0.02, *p* = 0.895, *η_p_*^2^ < 0.01, between the two groups (see Table 4).

### 3.4. Discussion of Study 2

The results of Study 2 showed that there were no significant differences between the experimental group and the control group in terms of average daily short-form video viewing time and preference for video content, which excluded the interference of previous video-viewing experience and preference for video content. Under the single-cue condition, there was no significant difference in PM accuracy between the experimental group and the control group, indicating that short-form videos do not significantly impair the performance of single-cue PM.

## 4. General Discussion

This study explored the impact of short-form videos on PM within the framework of the attention residue theory. From the perspective of the attention residue theory, the high-stimulation characteristics and frequent switching mode of short-form videos make individuals’ attention remain on the previous video content after viewing, forming continuous cognitive activation (Leroy, 2009; Chiossi et al., 2023). This occupied residual attentional resources make it difficult for individuals to allocate sufficient cognitive resources to cue identification and intention retrieval of PM tasks in subsequent tasks, ultimately resulting in a decrease in PM accuracy. The results of Study 1 showed that the PM accuracy of the experimental group was significantly lower than that of the control group, and this difference was not caused by the confusion of irrelevant variables such as the average daily short-form video viewing time and preference for video content between the two groups. This finding is consistent with the research conclusions of Chiossi et al. (2023) and Barton and Smyth (2025), further confirming the reliability of the negative impact of short-form videos on PM performance. At the same time, Study 1 divided PM into focal and non-focal types by manipulating cue focality, expecting short-form videos to only impair non-focal PM, which is more dependent on attention. However, the results showed that short-form videos significantly impaired the performance of both types of PM. The cue monitoring of non-focal PM is highly dependent on attention, while the cue monitoring of focal PM has a low dependence on attention (Harrington et al., 2025). According to the attention residue theory, short-form videos should impair non-focal PM performance to a greater extent. However, the results of this study are inconsistent with the views of the attention residue theory and the hypotheses of this study. This may be because the attention residue theory only explains that the content of short-form videos may occupy the attention of subsequent PM tasks, but does not indicate whether short-form videos also interfere with bottom-up stimulus identification. There is evidence that after watching short-form videos, individuals’ ability to identify stimuli also decreases (Walla & Zheng, 2024).

### 4.1. Theoretical Mechanisms Underlying PM Impairment

The performance of the ongoing task during PM intention maintenance can usually reflect changes in attention (Cohen et al., 2012). The results of Study 1 showed that the ongoing task reaction speed in the experimental group tended to be faster than that in the control group, indicating that short-video use reduces task processing time and attenuates attention consumption during the intention retention interval. The ongoing task, as a basic task, accounts for as high as 95% of the total trials, and its processing priority is usually high (Anderson et al., 2019). The PM task is a future-oriented task that requires us to reserve part of our attention to process the PM task. Therefore, the faster ongoing task reaction speed after viewing short videos prevents individuals from fully processing prospective memory tasks, thereby resulting in impaired PM performance.

### 4.2. Cue Number as a Key Moderator

To further reveal the relationship between the attentional demand of PM tasks and prolonged viewing of short-form videos, Study 2 further reduced the number of cues for PM tasks. A single PM cue is easy for forming an automatic connection between cues and behaviors (Einstein & McDaniel, 2010; Rothen & Meier, 2014), which can reduce the attentional consumption of PM tasks. The results of Study 2 showed that under the single PM cue condition, there was no significant difference in PM accuracy between the experimental group and the control group, indicating that watching short-form videos does not significantly interfere with single-cue PM tasks that have a relatively low dependence on attention. This further indicates that the impairment of PM by short-form videos is affected by attention. The attention residue theory holds that the impairment of short-form videos stems from the occupation of attentional resources by residual video content (Chiossi et al., 2023; Leroy, 2009). When the attentional demand of the PM task is reduced to a certain level, even if there is attention residue, the remaining attentional resources are sufficient to support the smooth execution of the task, so the impairment effect no longer exists. This result not only reveals the key regulatory role of the number of cues in the relationship between short-form videos and PM but also indirectly confirms the core logic of the attention residue theory, that is, the impairment of PM by short-form videos is essentially a result of resource competition. When the resources required by the task are reduced, the competition effect will be weakened. This verifies the view of the attention residue theory.

### 4.3. Limitations and Future Research Directions

While verifying the partial rationality of the attention residue theory, this study also expands the understanding of the cognitive mechanism underlying the impairment of PM caused by short-form video use: the impairment of PM by short-form videos is not a single attention residue effect, but may involve the joint action of multiple mechanisms such as attentional resource competition, decreased cognitive control ability, and reduced stability of intention representation. Future research needs to further separate the relative contributions of these mechanisms and construct a more comprehensive theoretical model. However, the existing research still has certain limitations. First, this study only set a single short-form video viewing duration (40 min) and did not examine the impact of different viewing durations (e.g., 10 min, 20 min) on PM, so it is impossible to determine the minimum viewing time for short-form videos to impair PM. Second, the participants in this study were all college students aged 19–25, who have relatively strong cognitive abilities and high acceptance of, and adaptability to, short-form videos. The attention ability of school-age children is easily interfered with (Wetzel et al., 2021), and their PM performance may be more easily affected by short-form videos. Third, this study only used behavioral responses to reflect changes in attention, but behavioral indicators have low sensitivity. Eye-tracking technology can reflect the time, object, and effort of attention in real-time, and has more advantages in reflecting changes in attention (Brunyé et al., 2019). Fourth, the traditional lab-based PM tasks used in the present study have relatively low ecological validity. Future research could design PM tasks with higher ecological validity in real-life settings and include both time-based and event-based PM tasks, thereby better capturing the impact of short-form video use on PM in everyday life. Finally, in this study, video content was selected based on participants’ interests; however, the detrimental effect of short-video use on PM may be influenced by video content. For example, individuals have better memory for negative stimuli (Hostler et al., 2018), so watching videos with negative content might leave a larger attention residue afterwards, potentially causing greater impairment in subsequent PM performance.

### 4.4. Theoretical and Practical Implications

The present study systematically investigated the impact of short-form video use on PM through two experiments, offering significant theoretical and practical implications. At the theoretical level, the findings partially support the attention residue theory, which posits that after prolonged viewing of short-form videos, individuals’ attention partially lingers on the previously viewed video content, thereby interfering with subsequent PM tasks. Notably, the results indicate that short-form videos impair not only non-focal PM—which is highly dependent on attention—but also focal PM. This suggests that the detrimental effect of short-form videos on PM is not limited to a unitary attentional resource competition mechanism but may additionally involve interference with cue identification processes, thereby extending the explanatory boundaries of the attention residue theory.

At the practical level, the study reveals that a single-cue condition attenuates the impairing effect of short-form videos on PM, carrying important implications for daily life. Since many real-life PM tasks involve a single cue, such tasks may be more resilient to the cognitive interference induced by short-form video use compared to tasks requiring monitoring of multiple cues. Therefore, the findings suggest that after watching short-form videos, individuals can reduce the risk of PM failure by associating to-be-remembered intentions with a single cue, thereby enhancing quality of life and safety in everyday situations. In learning environments, students often engage with short-form videos during breaks, and the subsequent decline in PM performance may lead to forgotten assignments, missed deadlines, or incomplete study plans. Educators and learners should be aware that even brief periods of short-form video consumption can impair the ability to remember future intentions, particularly when multiple cues are involved.

## 5. Conclusions

This study investigated the impact of short-form video use on PM through two experiments. The results showed that prolonged viewing of short-form videos significantly impairs the performance of both focal and non-focal PM, indicating that it simultaneously interferes with cue identification and attention monitoring. When the non-focal PM cue is simplified to a single one, the impairment effect of short-form videos is no longer significant, indicating that the impairment of PM by short-form videos depends on the attentional demand of the task, which partially supports the view of the attention residue theory. However, given that short-form videos impair both types of PM, their impact mechanism may not be limited to the occupation of attentional resources but also involves the process of interfering with cue identification.

## Figures and Tables

**Table 1:** The daily short-form video viewing time and preference for video content in Study 1 (*M* ± *SD*).

		Daily Viewing Time (min)	Preference for Video Content
Focal	Experimental	72 (34)	7.21 (1.56)
	Control	61 (30)	7.19 (1.39)
Non-focal	Experimental	74 (38)	6.89 (1.50)
	Control	65 (36)	6.72 (1.46)

Note. Experimental = experimental group, Control = control group.

**Table 2:** The PM task and ongoing task performance in Study 1 (*M* ± *SD*).

		PM Task		Ongoing Task	
		ACC	RT (ms)	ACC	RT (ms)
Focal	Experimental	0.60 (0.26)	1002 (324)	0.77 (0.09)	971 (273)
	Control	0.75 (0.28)	1055 (257)	0.76 (0.08)	1075 (181)
Non-focal	Experimental	0.34 (0.29)	1083 (410)	0.81 (0.08)	1047 (259)
	Control	0.48 (0.32)	1259 (411)	0.81 (0.07)	1118 (280)

**Table 3:** The daily short-form video viewing time and preference for video content in Study 2 (*M* ± *SD*).

	Daily Viewing Time (min)	Preference for Video Content
Experimental	63 (35)	7.21 (1.56)
Control	71 (33)	6.72 (1.46)

**Table 4:** The PM task and ongoing task performance in Study 2 (*M* ± *SD*).

	PM Task		Ongoing Task	
	ACC	RT (ms)	ACC	RT (ms)
Experimental	0.51 (0.34)	1192 (298)	0.79 (0.08)	1011 (254)
Control	0.60 (0.33)	1288 (295)	0.81 (0.09)	1019 (215)

## Data Availability

The raw data supporting the conclusions of this article will be made available by the authors upon request.

## Abbreviations

The following abbreviations are used in this manuscript:

PM	Prospective Memory
ACC	Accuracy
RT	Reaction Time
